# Retrospective analysis of nanoparticle albumin-bound paclitaxel combined with toripalimab and platinum as first-line treatment for Chinese patients with recurrent or metastatic head and neck cancer

**DOI:** 10.3389/fonc.2026.1674467

**Published:** 2026-02-26

**Authors:** Jinlan Li, Rui Zhong, Shuo Yang, Yi He, Wei Wang, Po Chen

**Affiliations:** 1 Department of Pharmacy the Affiliated Cancer Hospital of Xiangya School of Medicine, Central South University/Hunan Cancer Hospital, Changsha, China; 2 Department of Gastroenterology and Urology the Affiliated Cancer Hospital of Xiangya School of Medicine, Central South University/Hunan Cancer Hospital, Changsha, China

**Keywords:** nab-paclitaxel, toripalimab, R/M HNSCC, cisplatin, carboplatin, immunotherapy, first-line treatment

## Abstract

**Objective:**

To evaluate the clinical efficacy and safety of toripalimab combined with albumin-bound paclitaxel and cisplatin/carboplatin as first-line treatment for recurrent or metastatic head and neck squamous cell carcinoma (R/MHNSCC).

**Methods:**

Thirty-five patients with advanced R/MHNSCC admitted to Hunan Cancer Hospital (January 2021-December 2023) received first-line treatment with toripalimab plus albumin-bound paclitaxel and cisplatin/carboplatin. Efficacy was assessed using RECIST 1.1, and adverse events were evaluated according to NCI-CTCAE 5.0.

**Results:**

A total of 35 patients were assessed for efficacy, with partial response (PR) in 21 (60.0%), stable disease (SD) in 6 (17.1%), and progressive disease (PD) in 8 (22.9%). The overall response rate (ORR) was 60.0%, and the disease control rate (DCR) was 77.1%. The follow-up period concluded on August 10, 2024, with a median follow-up duration of 22.0 months (range: 15.0-27.0 months). Among the cohort, 8 patients experienced PD, and 7 patients succumbed to the disease; 13 patients continued treatment without disease progression. The median progression-free survival (PFS) for the entire cohort was 7.0 months (95% CI: 4.4-9.6 months), while the median overall survival (OS) had not yet been reached. Further results of Cox proportional hazards regression analysis showed that programmed death-ligand 1 (PD-L1) expression status was an independent risk factor for progression-free survival (PFS), whereas bone metastasis status and a history of surgery for head and neck squamous cell carcinoma were key risk factors for overall survival. The primary adverse reactions observed included bone marrow suppression, hypothyroidism, rash, neurotoxicity, pneumonia, and abnormal liver function. The majority of patients experienced grade I to II adverse reactions; however, one patient (4.3%) exhibited grade III adverse reactions, specifically an immune-related rash, and two patients experienced grade IV adverse reactions, one with leukopenia and the other with neutropenia. Notably, there were no deaths attributable to toxicity.

**Conclusion:**

Toripalimab combined with albumin-bound paclitaxel and cisplatin/carboplatin demonstrates promising efficacy with manageable adverse reactions as first-line treatment for R/MHNSCC. However, further research involving expanded sample sizes and randomized controlled trials is warranted to substantiate these findings.

## 1 Introduction

The initial presentation of head and neck squamous cell carcinoma (HNSCC) typically occurs in anatomically concealed regions, resulting in over 60% of patients being diagnosed at a locally advanced stage. Despite undergoing extensive treatment, 40% to 60% of these patients ultimately develop local recurrence or distant metastasis (1, 2), with a 5-year survival rate of less than 50% (3). At present, palliative chemotherapy constitutes the primary therapeutic approach for most recurrent and metastatic head and neck squamous cell carcinomas. A frequently employed first-line chemotherapy regimen includes the combination of cisplatin and 5-fluorouracil (PF regimen) or paclitaxel. Paclitaxel drugs are utilized as first-line and second-line treatments for locally advanced, recurrent, or metastatic head and neck malignancies. Albumin-bound paclitaxel is an innovative formulation that uses albumin as a carrier to enhance drug distribution within tumor cells, mitigate allergic reactions, and elevate local drug concentrations in tumors. Compared with conventional solvent-based paclitaxel, it does not require premedication and carries a lower risk of allergic reactions. While numerous studies have investigated the efficacy of albumin-bound paclitaxel in treating non-small cell lung cancer and breast cancer, there is a notable paucity of research concerning its application in advanced, recurrent, or metastatic head and neck tumors (3, 4).

Immune checkpoint inhibitors (ICIs) are extensively utilized in the management of recurrent and metastatic head and neck squamous cell carcinoma (R/MHNSCC). However, in my country, only nivolumab and pembrolizumab have received regulatory approval for the treatment of head and neck tumors. The high cost of these imported medications poses a significant barrier to widespread patient access. Furthermore, domestically produced immune checkpoint inhibitors have not yet been approved for the indication of R/MHNSCC. This study primarily investigates the efficacy and safety of toripalimab in combination with albumin-bound paclitaxel and cisplatin as a first-line treatment for recurrent and metastatic advanced head and neck squamous cell carcinoma in a cohort of 35 patients admitted to our hospital between January 2021 and December 2023. The findings are presented as follows.

## 2 Materials and methods

### 2.1 Patient population

Data were collected on 35 patients diagnosed with advanced head and neck squamous cell carcinoma (R/MHNSCC) exhibiting recurrence or metastasis, who received first-line treatment with a combination of toripalimab, albumin-bound paclitaxel, and cisplatin or carboplatin at our hospital between January 2021 and December 2023.

Inclusion criteria are as follows:

1. Age ranges from 18 (inclusive) to 75 years old.
2. Eastern Cooperative Oncology Group (ECOG) performance status score of 0 to 1.
3. Histologically confirmed diagnosis of squamous cell carcinoma.
4. Patients with Stage IVa-IVc disease have not undergone chemotherapy following recurrence.
5. Presence of at least one measurable lesion, as confirmed by imaging within 28 days prior to randomization, assessed according to RECIST 1.1 criteria (Note: bone metastasis or central nervous system [CNS] metastasis alone does not qualify as a measurable lesion).
6. Prior to treatment, the patient’s complete blood count, as well as liver and kidney function tests, are within normal limits, and there are no contraindications to chemotherapy.
7. The patient has undergone a minimum of two treatment courses and has completed at least one efficacy evaluation.

Exclusion criteria:

1. Patients with non-advanced or non-recurrent metastasis will be excluded.
2. The Paclitaxel albumin plus platinum regimen is not employed as the first-line treatment.
3. Preoperative neoadjuvant and induction radiotherapy will be excluded.
4. Patients diagnosed with nasopharyngeal carcinoma will be excluded.
5. Individuals with cardiovascular and cerebrovascular diseases, malignant tumors, or other severe illnesses will be excluded.
6. Patients with contraindications to systemic chemotherapy will be excluded.
7. Patients exhibiting language, cognitive dysfunction, or mental illness will be excluded.

This clinical study was approved by the Ethics Committee of Hunan Cancer Hospital.

### 2.2 Treatment options

All patient treatments were administered following consultations with a senior medical professional holding the title of Associate Chief Physician or higher. Consent for treatment was obtained through a signed authorization from either the patient or their designated representative. The immunotherapy administered to all study participants was integrated with other therapeutic regimens; no immunotherapy was administered in isolation. Specific drugs administered included: Teplizumab at a dosage of 240 mg on the first day; albumin-bound paclitaxel at 130 mg/m², delivered via intravenous drip on the 1st and 8th days; and either cisplatin at 75 mg/m² or carboplatin with an AUC of 5, administered intravenously on the first day. Each treatment cycle spanned 21 days. The medication regimen continued until disease progression or the onset of intolerable toxicity, with a maximum of six treatment cycles. Following the completion of the combined treatment course, patients who exhibited stable disease or better efficacy and demonstrated good tolerance were transitioned to maintenance therapy with immune checkpoint inhibitors in the immune group. Routine supportive treatments, including antiemetics and hepatoprotective agents, are administered. Hematological parameters, as well as hepatic and renal functions, are assessed before and after each chemotherapy session. Additionally, thyroid function and myocardial enzyme levels are monitored following each treatment cycle. In the event of bone marrow suppression, immediate supportive interventions, such as granulocyte colony-stimulating factor (G-CSF), are provided, and secondary preventive measures are implemented. Should grade 4 adverse reactions arise, the dosage of chemotherapeutic agents in subsequent cycles will be reduced by 25%.

### 2.3 Data collection and follow-up

Clinical data for patients were obtained via the medical record query system at Hunan Provincial Hospital. The dataset encompassed variables such as patient age, gender, tumor stage, primary tumor site, smoking history, alcohol consumption history, betel nut usage history, Performance Status (PS) score, PD1 expression levels, and the type of immune or chemotherapy treatment administered at the time of diagnosis. Patient follow-up was conducted through the hospital’s patient follow-up system, primarily utilizing outpatient visits and telephone consultations. The starting point of the study was that the patients were diagnosed with recurrent and metastatic advanced head and neck squamous cell carcinoma (R/MHNSCC).

### 2.4 Efficacy and adverse reaction evaluation

All patients underwent imaging examinations every 2–3 courses of treatment to evaluate efficacy. The efficacy evaluation was based on the Response Evaluation Criteria in Solid Tumors (RECIST) version 1.1, which was categorized into complete response (CR), partial response (PR), stable disease (SD), and progressive disease (PD). The objective response rate (ORR) was the percentage of evaluable cases with CR or PR. Patients with CR or PR were required to undergo imaging confirmation again after 4 courses of treatment; the disease control rate (DCR) was the percentage of evaluable cases with CR, PR, or SD. Progression-free survival (PFS) was defined as the time from the start of treatment with this regimen to the occurrence of disease progression. Adverse reactions during treatment were evaluated according to the NCI-CTC 5.0 standard.

### 2.5 Statistical methods

In our study, all statistical analyses were conducted in R (version 4.2.2) using the MATCHIT package, while additional analyses were performed in STATA (5). Categorical variables, including gender, primary tumor site, metastatic site, ECOG score, and the administration of surgery and radiotherapy, were described by frequency. Continuous variables, such as age, were summarized using the median. The Kaplan-Meier method was employed to estimate the median progression-free survival (PFS), and the Log-Rank test was utilized to compare survival rates between groups P<0.05 indicated that the difference was statistically significant.

## 3 Results

### 3.1 Patient baseline characteristics

The baseline characteristics of the 35 patients included in this study are presented in **Table 1**. The cohort had a mean age of 55 years (SD = 9), with a majority being male (85.7%). The most common primary tumor site was the tongue (51.4%), followed by the oral cavity (20.0%). Most patients were diagnosed with advanced or recurrent disease, with 48.6% presenting with Stage IV and 45.7% with recurrent cancer. Metastatic disease was prevalent, with cervical lymph node involvement observed in 62.9% of patients, while lung metastasis and supraclavicular lymph node metastasis were present in 22.9% each; 40.0% of patients had three or more metastatic sites. A history of smoking was reported by 68.6% of patients. Regarding performance status, 62.9% had an ECOG score of 1. PD-L1 expression was assessed in 20 patients (57.1% of the cohort), of whom 13 (37.1% of the total) had a Combined Positive Score (CPS) of 1 or greater. Prior treatments included surgery for head and neck squamous cell carcinoma in 60.0% of patients, whereas only 14.3% had a history of radiotherapy.

**Table 1:** Patient demographics and baseline characteristics.

Characteristic	N = 35	Characteristic	N = 35
Age		Other metastatic sites, n (%)	
Mean ± SD	55 ± 9	yes	16 (45.7%)
Median (Q1, Q3)	55 (46, 61)	no	19 (54.3%)
Primitive site, n (%)		Number of metastatic sites, n (%)	
Hypopharynx	3 (8.6%)	≥3	14 (40.0%)
Tongue	18 (51.4%)	<3	21 (60.0%)
Gums	3 (8.6%)	Smoking, n (%)	
Larynx	4 (11.4%)	Yes	24 (68.6%)
Oral cavity	7 (20.0%)	No	11 (31.4%)
Gender, n (%)		Drinking, n (%)	
Male	30 (85.7%)	Yes	16 (45.7%)
Female	5 (14.3%)	No	19 (54.3%)
Age stratification, n (%)		Areca, n (%)	
<60	23 (65.7%)	Yes	3 (8.6%)
≥60	12 (34.3%)	No	32 (91.4%)
Staging, n (%)		Performance score, n (%)	
Stage III	2 (5.7%)	0	12 (34.3%)
Stage IV	17 (48.6%)	1	22 (62.9%)
Recurrence	16 (45.7%)	2	1 (2.9%)
Mediastinal Metastasis, n (%)		Underlying disease, n (%)	
yes	3 (8.6%)	Yes	14 (40.0%)
no	32 (91.4%)	No	21 (60.0%)
Supraclavicular Lymph Node Metastasis, n (%)		History of HNSCC surgery, n (%)	
yes	8 (22.9%)	Yes	21 (60.0%)
no	27 (77.1%)	No	14 (40.0%)
Cervical lymph node metastasis, n (%)		History of radiotherapy, n (%)	
yes	22 (62.9%)	Yes	5 (14.3%)
no	13 (37.1%)	No	30 (85.7%)
Bone metastasis, n (%)		PD-L1, n (%)	
yes	5 (14.3%)	Not assessed	15 (42.9%)
no	30 (85.7%)	CPS<1	7 (20.0%)
Lung metastasis, n (%)		CPS≥1	13 (37.1%)
yes	8 (22.9%)		
no	27 (77.1%)		

### 3.2 Efficacy

**Figure 1:**
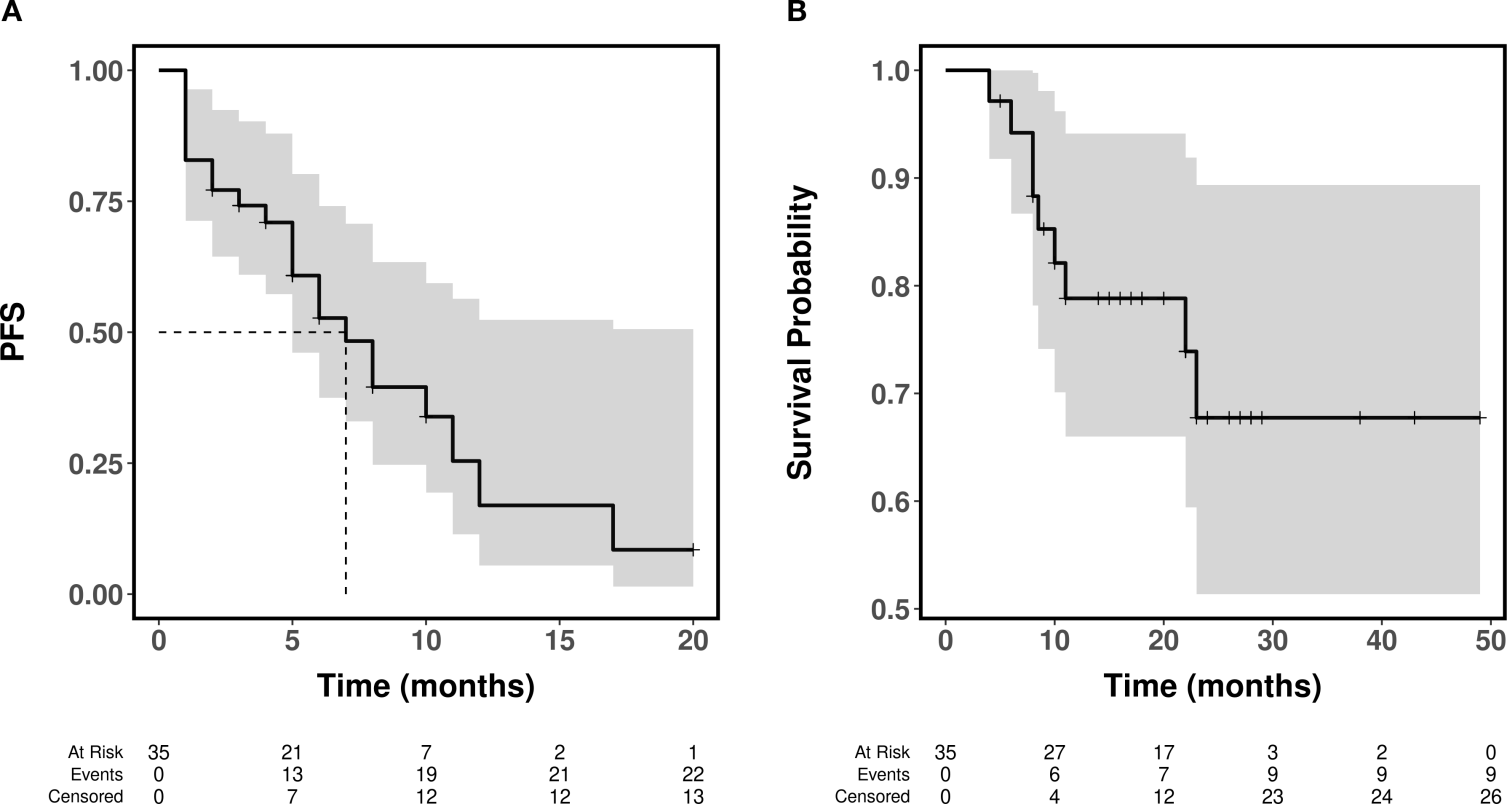
Kaplan–Meier curves for PFS and OS. **(A)** Kaplan–Meier curves for PFS; **(B)** Kaplan–Meier curves for OS.

Among the 35 patients, 15 cases (42.8%) completed the planned 6 cycles of combination therapy, while 9 cases (60.0%) discontinued treatment prematurely due to disease progression or adverse events. Specifically, 12, 8, and 15 patients received ≤3 cycles, 4–5 cycles, and ≥6 cycles of treatment, respectively. Objective efficacy was assessed for all patients, revealing that 21 patients (60.0%) achieved partial response (PR), 6 patients (17.1%) exhibited stable disease (SD), and 8 patients (22.9%) experienced progressive disease (PD). The overall response rate (ORR) was 60.0%, and the disease control rate (DCR) was 77.1%. The follow-up period extends until August 10, 2024, with a median follow-up duration of 22.0 months (range: 15.0 - 27.0 months). Among the cohort of 35 patients, 8 patients experienced disease progression (PD), and 7 patients succumbed to the disease. Thirteen patients continued their medication regimen without any signs of disease progression. The median progression-free survival (PFS) for the entire cohort was 7.0 months (95% CI: 4.4 to 9.6 months), while the median overall survival (OS) has not yet been reached. However, the 3-year survival rates for the cohort were 83.76%, 71.98%, and 71.98%, respectively. Detailed data are presented in **Table 2** and **Figure 1**.

**Table 2:** Response to immunotherapy in patients.

Best overall response	Example number	number (%)
CR	0	0.0
PR	21	60.0
SD	6	17.1
PD	8	22.9
ORR	21	60.0
DCR	27	77.1

CR, complete response; PR, partial response; SD, stable disease; PD, progressive disease; ORR, objective response rate (CR + PR); DCR, disease control rate (CR + PR + SD).

### 3.3 Factors affecting patient prognosis

**Figure 2:**
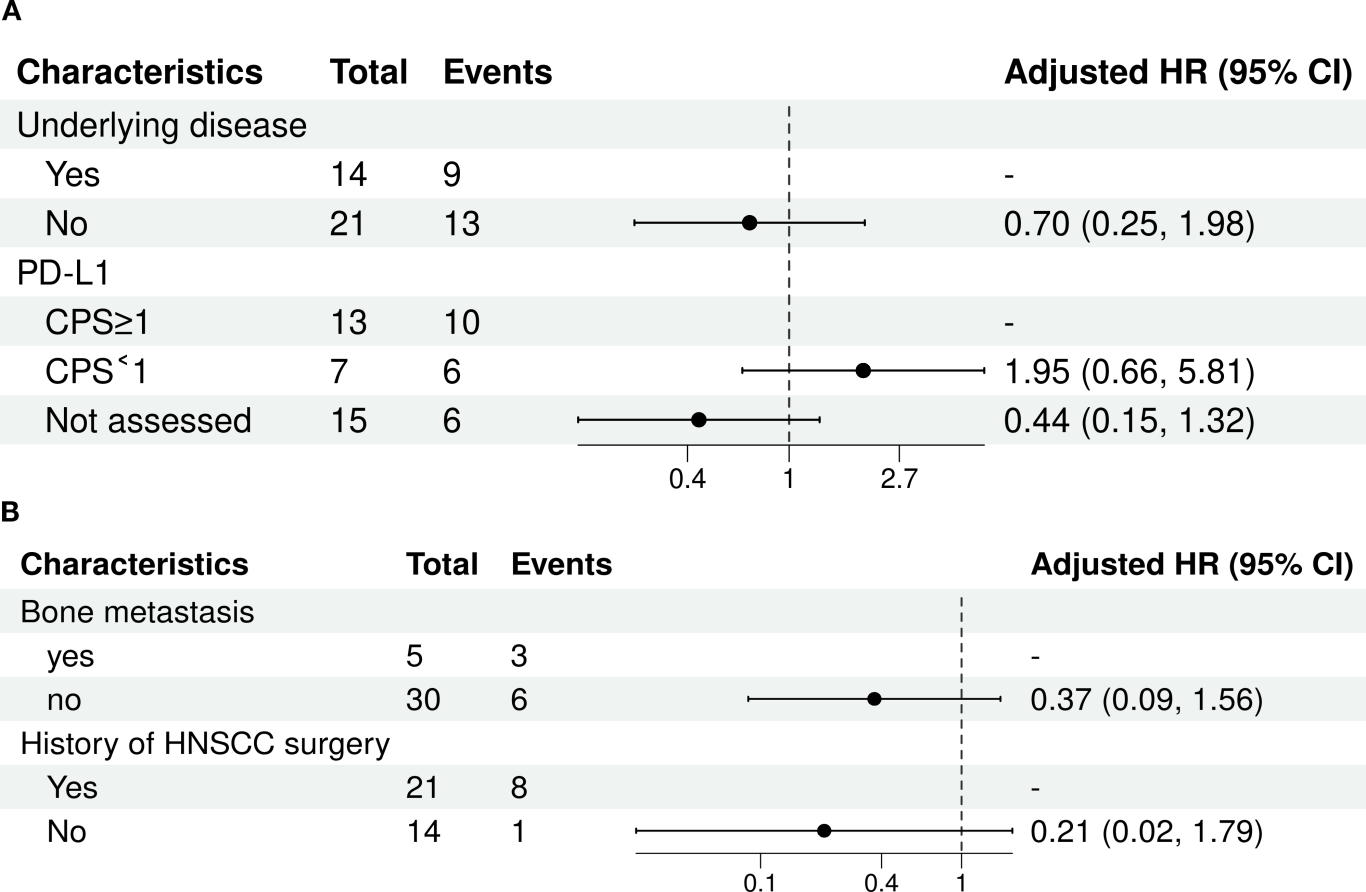
Multivariate analysis of influencing factors (Cox regression). **(A)** Multivariate Forest Plot of PFS; **(B)** Multivariate Forest Plot of OS.

A univariate survival analysis was conducted on a cohort of 35 patients. Progression-free survival (PFS) was associated with comorbidity status. As shown in **Figure 2**, multivariate analysis revealed significant associations between PD-L1 expression levels and mortality. Compared to patients whose PD-L1 status was not assessed (reference group), those with a CPS < 1 had a significantly higher risk of mortality (adjusted HR, 4.40; 95% CI, 1.19-16.24; p=0.026). Patients with a CPS ≥ 1 also showed an elevated, though not statistically significant, risk of mortality (adjusted HR, 2.26; 95% CI, 0.76-6.70; p=0.143). The presence or absence of an underlying disease was not significantly associated with mortality risk (adjusted HR for ‘No’ vs. ‘Yes’, 0.70; 95% CI, 0.25-1.98; p=0.501).

The results of the multivariate Cox proportional hazards analysis are presented in **Table 3**. Among patients with a history of bone metastasis, the presence of this condition was not significantly associated with mortality compared to its absence (adjusted HR 0.37; 95% CI, 0.09–1.56; p=0.175). Given the variable history of HNSCC surgery, patients without a surgical history did not show a statistically significant difference in mortality risk compared with those with a history of surgery (adjusted HR 0.21; 95% CI, 0.02–1.79; p=0.153).

**Table 3:** Univariate analysis of influencing factors (Cox regression).

Characteristic	PFS			OS		
	HR^1^	95% CI^1^	p-value	HR^1^	95% CI^1^	p-value
Primitive site						
Hypopharynx	—	—		—	—	
Tongue	0.62	0.17, 2.30	0.477	1.11	0.13, 9.46	0.925
Gums	0.24	0.02, 2.55	0.235	0.00	0.00, Inf	0.999
Larynx	1.14	0.22, 5.85	0.872	0.00	0.00, Inf	0.999
Oral cavity	0.81	0.17, 3.78	0.784	1.61	0.13, 19.37	0.707
Staging						
Stage III	—	—		—	—	
Stage IV	0.37	0.08, 1.73	0.206	4.01 ×10^07	0.00, Inf	0.999
Recurrence	0.54	0.12, 2.51	0.435	1.84 ×10^08	0.00, Inf	0.999
Mediastinal Metastasis						
Yes	—	—		—	—	
No	0.62	0.18, 2.14	0.450	0.71	0.09, 5.71	0.748
Supraclavicular Lymph Node Metastasis						
Yes	—	—		—	—	
No	0.88	0.35, 2.20	0.783	0.77	0.19, 3.09	0.711
Cervical lymph node metastasis						
Yes	—	—		—	—	
No	1.96	0.81, 4.72	0.134	1.64	0.44, 6.14	0.465
Bone metastasis						
Yes	—	—		—	—	
No	0.77	0.22, 2.67	0.683	0.23	0.06, 0.92	0.038*
Lung metastasis						
Yes	—	—		—	—	
No	1.16	0.41, 3.24	0.781	2.72	0.34, 21.83	0.347
Other metastatic sites						
Yes	—	—		—	—	
No	0.98	0.42, 2.32	0.971	3.15	0.65, 15.16	0.153
Number of metastatic sites						
≥3	—	—		—	—	
<3	0.78	0.33, 1.85	0.578	1.65	0.41, 6.63	0.479
Underlying disease						
Yes	—	—				
No	0.42	0.16, 1.11	0.079	2.05	0.42, 9.97	0.375
Gender						
Male	—	—		—	—	
Female	1.75	0.57, 5.33	0.324	1.56	0.32, 7.54	0.583
**Age**	1.02	0.97, 1.07	0.493	1.01	0.94, 1.08	0.814
Age stratification						
<60	—	—		—	—	
≥60	1.63	0.62, 4.24	0.318	1.12	0.28, 4.51	0.871
Smoking						
Yes	—	—		—	—	
No	0.80	0.33, 1.95	0.627	0.81	0.20, 3.29	0.771
Drinking						
Yes	—	—		—	—	
No	0.95	0.40, 2.23	0.902	1.07	0.29, 4.02	0.915
Areca						
Yes	—	—		—	—	
No	2.01	0.45, 8.93	0.357	0.90	0.11, 7.21	0.921
Performance score						
0	—	—		—	—	
1	0.80	0.31, 2.04	0.639	1.11	0.28, 4.48	0.881
2	2.09	0.25, 17.43	0.496	0.00	0.00, Inf	0.999
PD-L1						
Not assessed	—	—		—	—	
CPS<1	5.19	1.55, 17.38	0.008*	1.75	0.29, 10.54	0.540
CPS≥1	2.52	0.90, 7.09	0.079	1.68	0.37, 7.56	0.499
History of HNSCC surgery						
No	—	—		—	—	
Yes	1.03	0.44, 2.42	0.946	0.16	0.02, 1.28	0.084
History of radiotherapy						
No	—	—		—	—	
Yes	0.48	0.14, 1.65	0.243	1.85	0.23, 15.09	0.564

1*p<0.05; **p<0.01; ***p<0.001.

CI, Confidence Interval; HR, Hazard Ratio.

**Figure 3:**
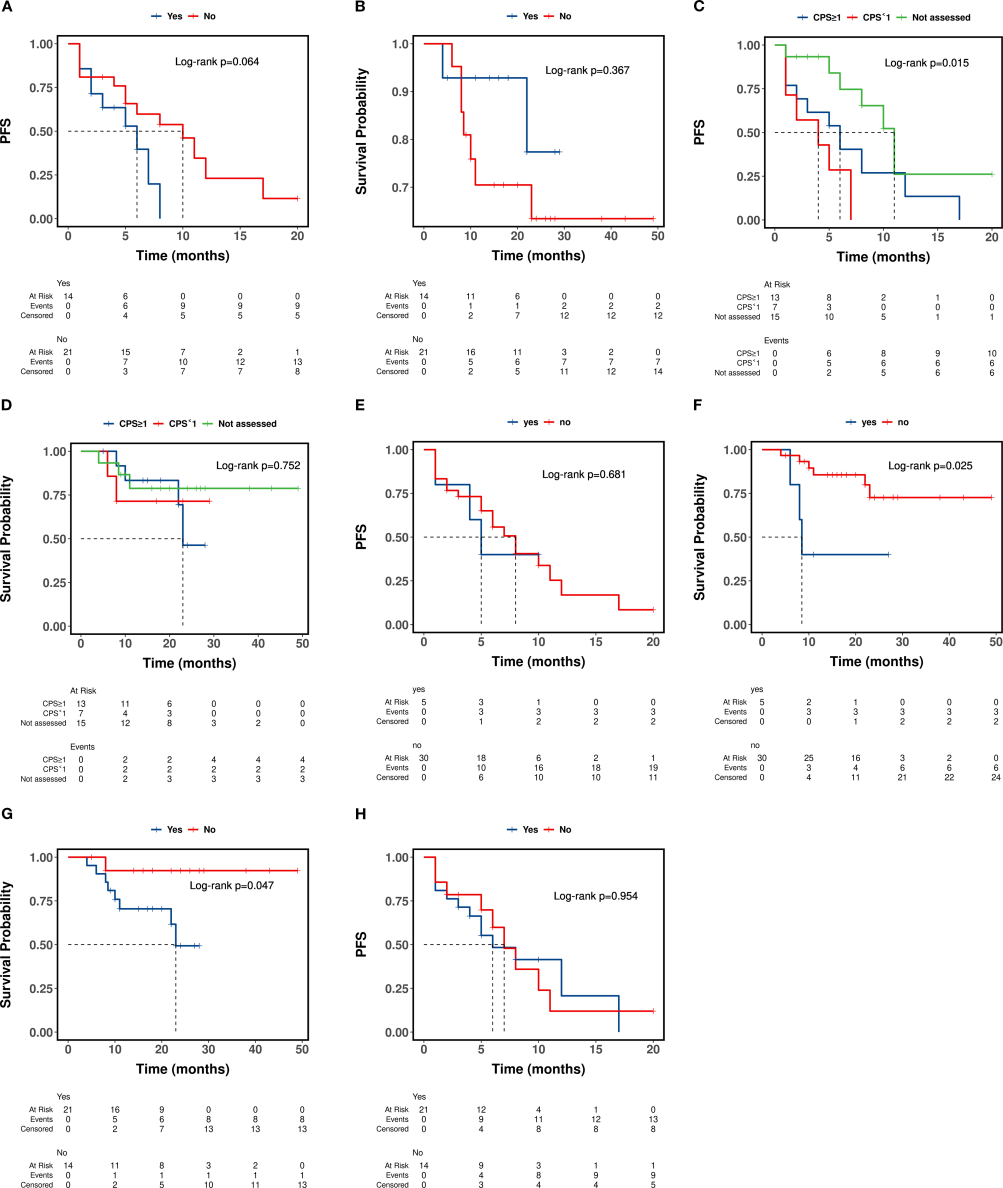
Impact of clinical variables on PFS and OS outcomes. **(A, B)** Kaplan-Meier Curves Stratified by Underlying disease; **(C, D)** Kaplan-Meier Curves Stratified by detection of PD-L1 expression; **(E, F)** Kaplan-Meier Curves Stratified by Bone Metastasis; **(G, H)** Kaplan-Meier Curves Stratified by History of HNSCC Surgery.

Conversely, variables such as gender, age, smoking status, alcohol consumption, history of betel nut use, and receipt of radiation therapy did not exhibit a statistically significant impact on either progression-free survival (PFS) or overall survival (OS) (**Table 3**, **Figure 3**).

### 3.4 Adverse events

The primary adverse reactions observed during treatment encompassed bone marrow suppression, hypothyroidism, rash, neurotoxicity, pneumonia, and liver function abnormalities, among others. Most patients experienced grade I to II adverse reactions; however, one patient (4.3%) exhibited grade III adverse reactions, specifically an immune-related rash. Additionally, two patients experienced grade IV adverse reactions: one patient presented with decreased white blood cell count, and another with reduced neutrophil count. Notably, there were no toxicity-related fatalities. The most frequently observed adverse reactions included neurotoxicity (26.1%) and hypothyroidism (21.7%). Additionally, loss of appetite (13.0%) and liver function impairment (13.0%) were reported, as outlined in **Table 4**.

**Table 4:** Adverse events.

Event Number of patients (%)	Any grade	Grade 1	Grade2	Grade3	Grade4
WBC count decreased (WBC)	2 (8.7)	0 (0.0)	1 (4.3)	0 (0.0)	1 (4.3)
Neutropenia	1 (4.3)	0 (0.0)	0 (0.0)	0 (0.0)	1 (4.3)
Hepatic injury	3 (13.0)	0 (0.0)	3 (13.0)	0 (0.0)	0 (0.0)
Hypothyroid	5 (21.7)	0 (0.0)	5 (21.7)	0 (0.0)	0 (0.0)
Eczema	1 (4.3)	0 (0.0)	0 (0.0)	1 (4.3)	0 (0.0)
Cardiac dysfunction	1 (4.3)	0 (0.0)	1 (4.3)	0 (0.0)	0 (0.0)
pulmonitis	1 (4.3)	0 (0.0)	1 (4.3)	0 (0.0)	0 (0.0)
loss of appetite	3 (13.0)	0 (0.0)	3 (13.0)	0 (0.0)	0 (0.0)
sensory neuropathy	6 (26.1)	0 (0.0)	6 (26.1)	0 (0.0)	0 (0.0)

## 4 Discussion

In recent years, the incidence of head and neck squamous cell carcinoma (HNSCC) has been on the rise. In 2018, approximately 700,000 new cases were reported, along with about 350,000 deaths (6). At the time of diagnosis, nearly 70% of patients had progressed to an advanced stage, with tumor cells extensively invading surrounding tissues. The efficacy of surgical treatment alone is suboptimal (7). Patients with locally advanced HNSCC have a poor prognosis, with more than 50% experiencing local recurrence or metastasis (8). Clinical studies have demonstrated that combination chemotherapy offers superior survival benefits for patients with advanced head and neck squamous cell carcinoma, effectively delaying tumor progression and extending survival time (9). Albumin-bound paclitaxel represents a novel cytotoxic anti-cancer agent. Utilizing nanotechnology, hydrophobic paclitaxel is conjugated with albumin to form nanoparticles approximately 130 nm in diameter. The unique transport mechanism of albumin enhances drug pharmacokinetics and augments anti-tumor efficacy (10). Research conducted by Chen LJ et al. (11). demonstrated that the combination of albumin-bound paclitaxel and nedaplatin in the treatment of advanced head and neck squamous cell carcinoma significantly prolongs patients’ progression-free survival (PFS) and overall survival (OS), while also enhancing their quality of life. Currently, Head and Neck Squamous Cell Carcinoma (HNSCC) is recognized as a highly immuno-invasive tumor type. Qiao et al. (12) have reported that Programmed Death-Ligand 1 (PD-L1) expression can be detected in approximately 50% to 70% of HNSCC cases, thereby providing a theoretical foundation for immunotherapy approaches. According to the guidelines established by the Chinese Society of Clinical Oncology (CSCO), the PD-1 inhibitors pembrolizumab and nivolumab are recommended as first-line treatments for relapsed or metastatic HNSCC. However, the overall survival rates associated with these treatments remain suboptimal. Additionally, both pembrolizumab and nivolumab are imported drugs in our country, rendering them costly and limiting their widespread accessibility. Consequently, there is an urgent need for treatment options that are low-toxicity, effective, and economically feasible.

In our study, the objective response rate (ORR) for the entire cohort of patients was 60.0%, with a median progression-free survival (PFS) of 7.0 months (95% CI: 4.4 to 9.6 months), and the median overall survival (OS) has not yet been reached. When compared to the real-world study conducted by Takashi Matsuki et al. (13). In Japan, where the ORR was 48% and the median PFS was 3.7 months, our study demonstrates superior outcomes. Additionally, the 3-year survival rates in our study were 83.76%, 71.98%, and 71.98%, respectively. It is important to acknowledge that this study is retrospective in nature and should be viewed as a hypothesis-generating analysis rather than a definitive efficacy trial. While our observed ORR of 60.0% and DCR of 77.1% are encouraging, these results must be interpreted with caution. Unlike landmark randomized controlled trials (RCTs) such as KEYNOTE-048 (14), which established the standard of care for R/M HNSCC under strictly controlled conditions, our real-world data reflects a more heterogeneous patient population and lacks a control arm. Therefore, any descriptive comparisons with RCT data are intended solely for contextualizing our observations within the current treatment landscape and do not imply non-inferiority or superiority.

The study conducted by Geoffrois et al. (15) corroborated the finding that elevated PD-L1 expression correlates with greater immunotherapy benefit, underscoring its substantial clinical utility in guiding therapeutic decisions. Similarly, research by Ferris et al. (16) on patients with recurrent and metastatic head and neck squamous cell carcinoma (HNSCC) demonstrated that PD-L1 inhibitors were significantly more effective in PD-L1-positive patients. Notably, the 2-year survival rate in this cohort nearly tripled to 16.9%, compared to 6.0% in the standard treatment group. Additionally, the median survival time was 7.7 months, compared with 5.1 months in the standard treatment group. In our study, Cox’s one-factor regression analysis indicated that PD-L1 expression may be an independent predictor of progression-free survival (PFS), although it did not reach statistical significance for overall survival (OS). Conversely, Bone metastasis exerts an impact on patients’ overall survival (OS); studies have demonstrated that bone metastasis is typically associated with significant morbidity and mortality (17). Clinically, bone metastasis can induce a series of severe skeletal-related events (SREs), including severe pain, pathological fractures, bone lesions requiring radiotherapy or surgical intervention, spinal cord compression, and malignant hypercalcemia (18, 19). These complications not only severely impair patients’ physical function and quality of life (20). But more importantly, substantial clinical evidence indicates that bone metastasis is an independent poor prognostic factor that predicts a significant reduction in patients’ overall survival (OS) (21–23).

Regarding safety, our study demonstrated that most patients experienced grade I to II adverse reactions. Specifically, one patient (4.3%) encountered a grade III adverse reaction, characterized by an immune-related rash. Additionally, two patients experienced grade IV adverse reactions: one patient exhibited a decrease in white blood cells, and another in neutrophils. Importantly, there were no toxicity-related fatalities. The incidence of adverse reactions observed in our study aligns with previously reported literature. Notably, thyroid dysfunction is frequently cited as a common adverse reaction to immunotherapy. In this study, thyroid dysfunction was observed in 5 patients (21.7%), a finding consistent with the literature (24). The primary mechanism of pathogenesis involves PD-1 inhibitors binding to PD-L1 ligands on tumor cells, thereby facilitating T cell activation. This process, while effectively targeting and destroying tumor cells, also damages the human endocrine system. Consequently, this can lead to thyroid dysfunction, pituitary abnormalities, and other endocrine-related disorders (25). Simultaneously, our study observed 6 cases (26.1%) of neurotoxicity-related adverse reactions, potentially attributable to the formulation structure of the albumin-paclitaxel-conjugated drug. The use of albumin-conjugated paclitaxel has been shown to increase the effective drug concentration in tumor cells by up to 33%. More importantly, it exhibits superior tissue penetration compared to traditional drugs; however, its associated neurotoxicity is also more pronounced (26–28). Our research findings indicate a lower incidence of treatment-related adverse events than reported in the literature (29). Specifically, when juxtaposed with the safety data from the KEYNOTE-048 study, our study demonstrated a reduced occurrence of adverse events, particularly those of Grade 3–4 severity or higher (11.5% vs. 52%). These results suggest that our research protocol is both safe and effective.

Several limitations must be addressed. First, the small sample size (n=35) from a single center limits the statistical power and the generalizability of our findings. Second, PD-L1 expression status, a critical biomarker for immunotherapy response in HNSCC, was missing for a significant portion of our cohort due to the retrospective nature of the data collection; this precludes a more granular analysis of which subgroups benefit most from the toripalimab combination. Third, inherent confounding factors—including variations in prior treatment history, tumor burden, and physician bias in treatment selection—may have influenced the clinical outcomes. Future prospective, large-scale randomized trials are required to validate the synergistic potential of nab-paclitaxel and toripalimab in this setting.

In conclusion, for patients with advanced or recurrent and metastatic head and neck squamous cell carcinoma (R/MHNSCC), particularly those at stages IVA and IVB, frontline immunotherapy in combination with chemotherapy has demonstrated efficacy in downstaging the disease and reducing tumor burden. This therapeutic approach facilitates subsequent conversion therapy or radiotherapy, thereby enhancing the potential for achieving a radical cure. Simultaneously, the therapeutic model involving the combination of teriplizumab with albumin-bound paclitaxel and platinum-based drugs offers a novel reference for the treatment of recurrent/metastatic head and neck squamous cell carcinoma (R/MHNSCC) in our country. This regimen demonstrates superior efficacy and safety compared to the currently approved pembrolizumab and nivolumab, while also being cost-effective and highly accessible. However, the present findings are limited to short-term efficacy data, and further investigation is needed into long-term outcomes and subsequent treatment protocols. Comprehensive studies with larger sample sizes are required to substantiate these preliminary results. The center will continue to conduct follow-up studies and monitor patient outcomes.

## Data Availability

The datasets presented in this study can be found in online repositories. The names of the repository/repositories and accession number(s) can be found in the article/supplementary material.
